# Illuminating Dersimelagon: A Novel Agent in the Treatment of Erythropoietic Protoporphyria and X-Linked Protoporphyria

**DOI:** 10.3390/ph17010031

**Published:** 2023-12-25

**Authors:** Katelyn E. Madigan, Sean R. Rudnick, Matthew A. Agnew, Numra Urooj, Herbert L. Bonkovsky

**Affiliations:** 1 Section on Gastroenterology & Hepatology, Department of Medicine, Wake Forest University School of Medicine, Winston-Salem, NC 27157, USA; srudnick@wakehealth.edu (S.R.R.); hbonkovs@wakehealth.edu (H.L.B.); 2 Department of Internal Medicine, Wake Forest University School of Medicine, Winston-Salem, NC 27157, USA; matagnew@wakehealth.edu; 3 Department of Medicine, Parkview Health, Fort Wayne, IN 46845, USA; numra009@gmail.com

**Keywords:** erythropoietic protoporphyria, X-linked protoporphyria, oral, dersimelagon

## Abstract

Erythropoietic protoporphyria (EPP) is a genetic disorder stemming from reduced ferrochelatase expression, the final enzyme in the pathway of heme biosynthesis. A closely related condition, X-linked protoporphyria (XLP), bears similar clinical features although it arises from the heightened activity of δ-aminolevulinic acid synthase 2 (ALAS2), the first and normally rate-controlling enzyme in heme biosynthesis in developing red blood cells. Both of these abnormalities result in the buildup of protoporphyrin IX, leading to excruciating light sensitivity and, in a minority of cases, potentially fatal liver complications. Traditionally, managing EPP and XLP involved sun avoidance. However, the emergence of innovative therapies, such as dersimelagon, is reshaping the therapeutic landscape for these conditions. In this review, we summarize salient features of the properties of dersimelagon, shedding light on its potential role in advancing our understanding of treatment options for EPP and XLP.

## 1. Introduction

Porphyrias constitute a diverse array of metabolic disorders, each linked to specific disruptions in one of the eight essential steps of heme production. These disorders can be broadly classified as hepatic or erythropoietic, indicating whether the accumulation of porphyrin precursors or porphyrins primarily occurs in the liver or the bone marrow. Additionally, a clinical classification into acute, blistering cutaneous and non-blistering cutaneous forms is valuable [1,2,3,4,5,6,7]. The cutaneous porphyrias, as well as acute hepatic porphyrias that may present with cutaneous manifestations, are summarized in Table 1. Familiarity with the clinical manifestations of various subtypes is important due to the ongoing study and optimization of much-needed therapeutic options.

Erythropoietic protoporphyria (EPP) is due to biallelic mutations in the FECH gene, which encodes for the enzyme responsible for the last step in heme biosynthesis, wherein PPIX is chelated with iron to form heme [5]. While approximately 4% of EPP patients carry two uncommon pathogenic FECH variants, the typical molecular abnormality found in around 96% of EPP patients involves a rare pathogenic FECH variant alongside a common intronic FECH variant known as c.315-48T>C (also historically referred to as IVS3-48T>C). This common intronic variant, known as the hypomorphic allele, has been shown to enhance the utilization of an abnormal splice site [8,9]. The missense or nonsense mutation on the other allele typically does not exhibit residual activity. However, when coupled with the other mutated allele, this leads to a 75% or greater decrease in FECH activity—causing an accumulation of PPIX in late erythroblasts with clinically evident disease [9]. An association between elevated PPIX and increased light sensitivity has been reported. As outlined in a study “patients with EPP with more severe phototoxicity (symptoms within 10 min of exposure) had significantly higher median ePPIX levels compared with those reporting symptoms after 10 min of sun exposure (2233 µg/dL [IQR, 1522–3056 µg/dL] vs. 1524 µg/dL [IQR, 1003–2002 µg/dL]; *p* < 0.001).” [10]. Other factors, such as innate skin pigmentation, also contribute to differences in light sensitivity.

Beyond the classic cutaneous manifestations, up to 47% of patients with EPP are found to have iron deficiency anemia. Microcytic and hypochromic anemia is due in part to a relative deficiency in the insertion of iron molecules into PP due to a deficiency in ferrochelatase activity. Nevertheless, patients with EPP, especially women, often have lower ferritin and transferrin saturations, even in the absence of abnormal blood loss.

Still, the administration of iron to such patients is often more harmful than beneficial in EPP but may help in XLP [4,5]. Though less common, progressive cholestatic liver disease may develop in 2–5% of patients, ultimately resulting in liver failure.

X-linked protoporphyria (XLP), a condition closely associated with the overproduction of protophyrin IX [PPIX], is associated with a variety of gain-of-function mutations, mainly deletions in exon 11, that result in increased δ-aminolevulinic acid synthase 2 (ALAS2), the enzyme responsible for the initial step in erythroid heme biosynthesis [8,9]. Located on the X chromosome, XLP was previously described as an X-linked dominant trait with essentially 100% penetrance, but there is recent evidence of heterozygous females who show varying phenotypic and biochemical heterogeneity, reflecting the random varying degree of X-chromosomal inactivation of the X chromosome that carries the mutant gene [8]. In EPP/XLP, blue light (peak effect at 410 nm, the Soret band) is absorbed by PPIX, which is excited to a singlet electron state; as it returns to the lower-energy triplet state, it emits energy that leads to increased oxidative stress and pro-inflammatory cascades and cytokines. These, in turn, can lead to pain, edema, and inflammatory responses [11,12].

Erythropoietic protoporphyria (EPP) and X-linked protoporphyria (XLP) are rare genetic photodermatoses with an estimated prevalence between 1.0 to 2.7 in 200,000 among the Caucasian population; however, the frequency of pathogenic ferrochelatase (FECH) mutations in the UK Biobank database suggests the potential for a higher prevalence of approximately 11.8 in 200,000 [13,14,15]. Specifically, within the pediatric population, EPP and XLP are currently believed to be the most common forms of porphyria [14,15]. Ongoing study continues to elucidate inherited and acquired porphyrias and their incidences [16].

When EPP or XLP is suspected, testing first involves the measurement of total erythrocyte protoporphyrin levels. Elevated levels of fractionated erythrocyte metal-free and zinc protoporphyrin help distinguish between EPP and XLP. Metal-free protoporphyrin is typically greater than 90% in EPP and ranges between 50–85% in XLP [10]. In XLP, larger concentrations of zinc-PPIX are typically observed in erythrocytes, likely related to the excess PPIX, to the adequacy of Zn, and to the normal activity of FECH, which is capable of inserting Zn, as well as Fe, into PPIX. Because plasma porphyrin levels may be normal or increased in EPP or XLP, recent guidelines do not recommend the use of plasma porphyrins alone to establish or rule out the diagnosis of EPP or XLP [17]. Nevertheless, the finding of an increase together with a fluorescence emission pattern showing a peak at 634 nM are useful in differential diagnosis. In contrast, in most other cutaneous porphyrias the emission peak occurs at ~620 nM, except for variegate porphyria, in which the peak emission is at 626 nM; the latter has proved helpful in diagnostic algorithms [4,5].

Studies are ongoing to expand the therapeutic options for these conditions, with sun avoidance previously used as the mainstay of therapy. Herein, we focus on a therapy that utilizes the activation of the melanocortin 1 receptor that has been found to exert anti-inflammatory effects [18]. This review serves to provide increased awareness and understanding of dersimelagon as a novel oral agent that has shown clinically meaningful and statistically significant benefits in phase 2 clinical trials, demonstrating its safety and efficacy in the treatment of EPP/XLP.

## 2. Previous Therapies

Behavioral modification is typically advised as first-line treatment, such as sun avoidance, use of zinc oxide sunblock, and protective clothing. However, this is insufficient due to the infeasibility of sun avoidance and indoor exposure to damaging wavelengths of sunlight passing through windows or arising from artificial light sources. Additionally, most sunscreens are more protective against lower wavelength UV radiation and not against the frequencies that cause EPP symptoms [19]. Narrowband ultraviolet B phototherapy has been attempted as a prophylactic measure in EPP. There were proposals for patients with EPP/XLP to purposely experience controlled and gradually increasing UVB radiation to produce skin thickening with increased melanin production. This controlled form of UVB radiation requires administration by a trained professional and may be inconvenient for patients, as it involves exposure at least three times weekly for at least five weeks. A small European study with 12 patients noted that it was ineffective in five of these patients, not meeting any statistical or clinical significance [20]. Recent EPP Management Guidelines still advise patients to wear opaque clothing and utilize light filters when feasible to minimize phototoxic symptoms [17].

Subsequently, there has been a treatment goal to induce eumelanogenesis without exposure to UV radiation. Early studies of beta-carotene as a treatment option necessitated daily high doses (a minimum of 180 mg/day for adults) for at least three months to reach carotenoid blood levels of at least 800 μg/dL [21,22,23]. Evaluation of these studies found data suggesting that the beta-carotene treatment efficacy was contradictory and that efficacy inversely correlated with study quality [24]; that is, a study was unable to find a significant difference between beta-carotene and placebo for photosensitivity in patients with EPP [25]. As reviewed in recent guidelines, previous therapies including, although not limited to, cimetidine, isoniazid, or pyridoxine, were not found to have clear benefits [17]. Additionally, there has not been clinical success with the use of vitamin C or N-acetyl cysteine for the treatment of EPP [26,27,28]. 

Treatment with cimetidine has been purported to inhibit delta-aminolevulinic acid [ALA] synthase; its use has therefore been proposed to reduce PPIX levels in patients with EPP. Though data are limited to case reports or small series without placebo controls or double-blind designs, there are some suggestions that photosensitivity may be decreased with cimetidine treatment [2]. An ongoing phase 2, double-blind, placebo-controlled cross-over trial of cimetidine is currently underway in the United States (NCT05020184).

Bitopertin is another oral agent undergoing a phase 2 clinical trial for the treatment of EPP (NCT05308472). As an oral selective glycine transport inhibitor, this agent restricts glycine uptake into erythroid cells. Glycine and succinyl-CoA are the substrates for ALA synthase, the first and rate-controlling enzyme of the PPIX and heme biosynthetic pathway. Afamelanotide (Scenesse, Clinuvel Pharma) was found to provide benefits in EPP and XLP due to its ability to increase eumelanin production by melanocytes, with an expected reduction in the severity of phototoxic reactions to sunlight or other strong light. Afamelanotide has little effect, if any, on the ongoing overproduction of PPIX [29]. It was approved in Europe by the European Medicines Agency and in the US by the FDA in 2019 as the first effective medical treatment for EPP [30,31]. Afamelanotide, [Nle4, D-Phe7]-a-MSH], is an analog of a-MSH, which stimulates the production of eumelanin as a non-selective agonist of melanocortin receptors. It has higher potency and stability than the natural MSH peptide. A recent observational animal study from Switzerland suggests the occurrence of a dose-dependent protective effect from liver damage related to EPP [32]. Afamelanotide is administered as a subcutaneous implant, which is injected every two months and gradually releases the active peptide. This treatment was found to reduce severe phototoxic reactions and improve quality of life; however, a trained professional is needed to administer the treatment. Nausea and loss of appetite are the most notable side effects, which may be due to the molecule’s ability to activate other MCRs non-selectively [33,34,35]. In May 2023, a study outlined challenges with obtaining afamelanotide for EPP or XLP [36]; notably, there are concerns regarding patient accessibility (Clinuvel Pharma is currently supplying afamelanotide to only a few selected centers) and costs (currently prices in the US are ~$55,000/implant) [36]. Thus, its use has been restricted to relatively few patients with EPP/XLP.

**Figure 1:**
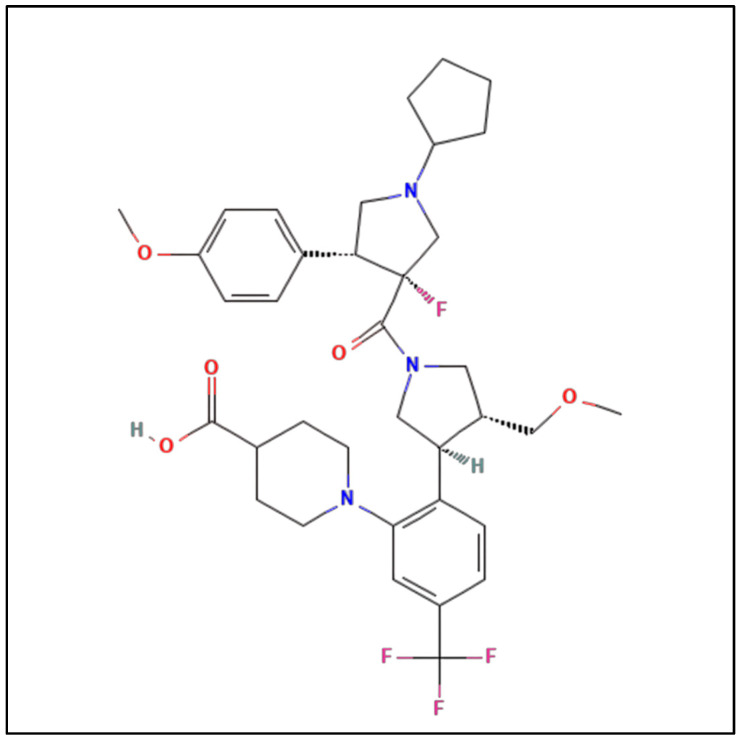
Chemical structure of Dersimelagon [18].

Dersimelagon (Figure 1) is an orally active small-molecule selective melanocortin-1 receptor agonist that increases the production of eumelanin, resulting in increased skin pigmentation and anti-inflammatory effects, resulting in increased duration of symptom-free sunlight exposure.

## 3. Metabolism and Pharmacokinetics of Dersimelagon

A key component of the appeal of dersimelagon is its oral bioavailability. Mitsubishi Tanabe Pharma Corporation has developed modification methods to improve its stability as a non-peptide [37]. Its molecular structure (Figure 1 [18]) has an extensive terminal modification to strengthen and protect the molecule from rapid degradation before reaching its target receptors. In contrast, afamelanotide is unsuitable for oral administration due to the liability of its breakdown in the small intestine by proteases and peptidases, as well as its large size, impairing absorption from the GI tract [37,38].

The molecular stability of dersimelagon was examined, with an assessment of the oral bioavailability and pharmacokinetics of dersimelagon tablets under a variety of gastric conditions such as a fed state, fasting state, acidic beverage consumption, using high-fat meals, and with a proton pump inhibitor (PPI) [39]. The 50-participant study was an open-label, multicenter, randomized, two-cohort, sequential, and cross-over study in which participants were randomized into two cohorts to receive 300 mg or 100 mg tablets under three gastric conditions: fasted (10+ h), fed (high-fat breakfast with and without esomeprazole 40 mg 2 h before breakfast) with water or a caffeine-free acidic carbonated beverage (355 mL of Canada Dry© ginger ale at a pH of 2.8). No effect was observed on the overall exposure following consumption of a high-fat meal, and Cmax was higher (22%, 90% confidence interval (CI) 1.05–1.42) in a fed state compared with fasted conditions. Similarly, the overall exposure AUC of dersimelagon was comparable following administration alone or in combination with esomeprazole; however, coadministration of esomeprazole led to a slight decrease in Cmax (fasted: 9%, 90% CI 0.77–1.07; fed: 24%, 90% CI 0.66–0.88) compared with the administration of dersimelagon alone. In general, the consumption of an acidic beverage increased the time to Cmax regardless of fed or fasted status and decreased the overall exposure to AUC and Cmax of dersimelagon [39].

**Figure 2:**
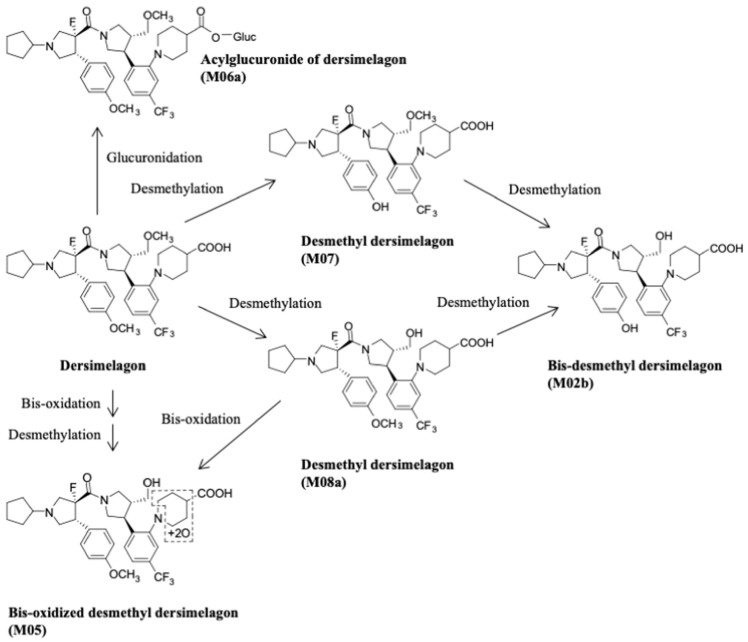
Major metabolites detected in human feces and plasma following oral administration of Dersimelagon in healthy adults and postulated metabolic pathways [18].

**Figure 3:**
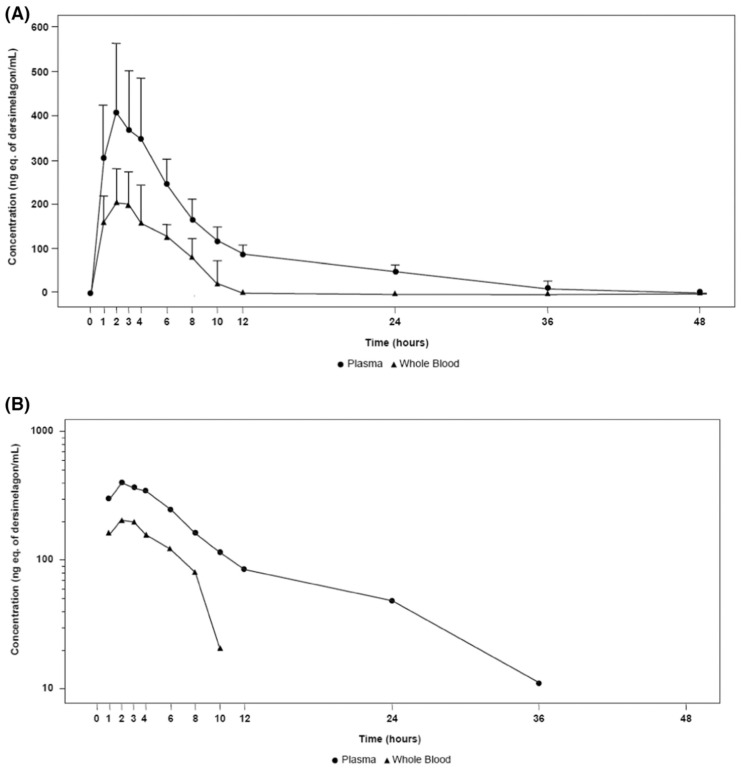
Mean total radioactivity concentration (ng. eq of Dersimelagon/mL) in plasma and whole blood shown in the first 48 h on (**A**) Linear scale with SD and (**B**) Semi-algorithmic scale, following oral administration in healthy adults [18].

The absorption, metabolism, and excretion of dersimelagon in rats, monkeys, and six humans (white males aged 30–65 years, weights 60–110 kg, and with BMI of 18–32 kg/m^2^, who were overall healthy and free from illness or disease) has been evaluated [18]. Participants received a single oral 100 mg dose of radioactive [^14^C] dersimelagon. Blood, urine, and fecal samples were collected periodically to evaluate Cmax, Tmax, AUC, apparent t^1/2^, and terminal elimination rate constant (K_el_). The median Tmax was two hours in humans (Table 2) [18]. As shown in Figure 2 [18], dersimelagon is extensively metabolized to glucuronide in the liver, which is eliminated in the bile. In the intestine, some of the glucuronide is metabolized back to the parent drug. In humans, elimination of radioactivity in urine was negligible (excretion of radioactivity into the urine: 0.31% of dose), and the primary route of excretion was feces, with more than 90% of the radioactivity recovered through five days post-dose. Based on these findings, it was concluded that a significant amount of radioactivity from [^14^C] dersimelagon was not retained in the human body, as shown below (Figure 3 adopted from Suzuki with the axes font increased for improved visibility while maintaining the integrity of the primary literature) [18].

## 4. Mechanism of Action

A mechanism of photoprotection is provided through the increased production of eumelanin, a black-brown pigment generated by melanocytes, keratinocytes, monocytes, endothelial cells, and fibroblasts. Eumelanin produced by melanocytes is influenced by five known distinct melanocortin receptors. Dersimelagon has the highest affinity for the melanocortin-1 receptor (MC1R), a G-protein coupled receptor on melanocytes that binds to melanocortin to regulate skin and hair color (Table 3) [40,41,42].

**Figure 4:**
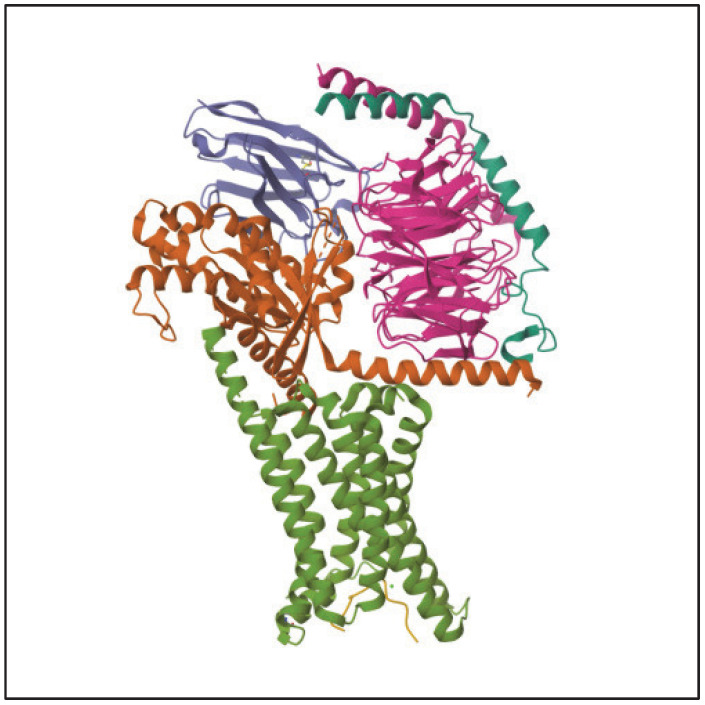
Cryo-EM structure of alpha-MSH-bound melanocortin-1 receptor in complex with Gs protein. Protein data bank of the National Science Foundation of the National Institutes of Health under grant R01GM1133198. https://www.rcsb.org/3d-view/7F4D/1, accessed on 23 July 2023.

**Figure 5:**
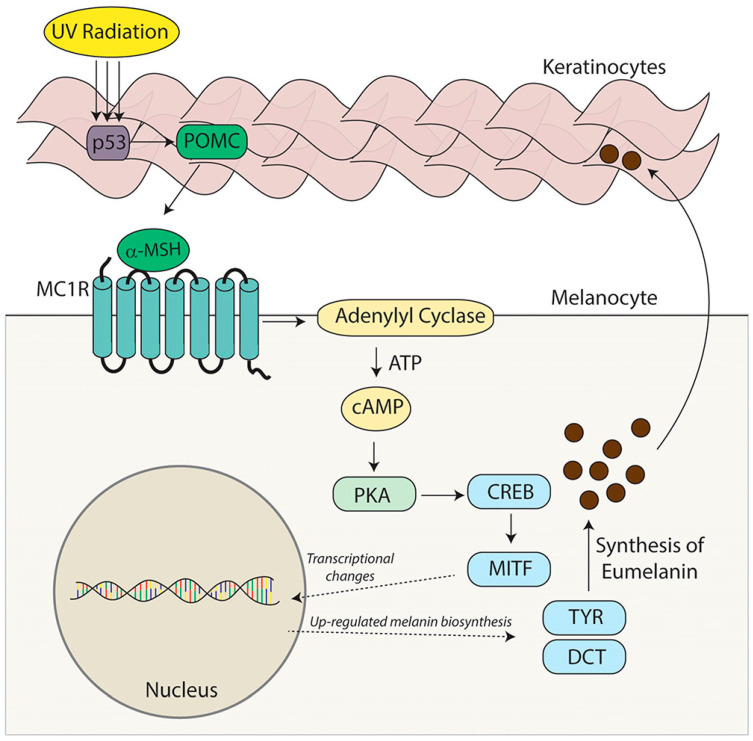
Model of predicted binding of Dersimelagon to MCR1 and the cascade of downstream effects leading to increased eumelanin production [41].

MC1R is established as the main driver of human pigmentation [34]. Potent peptide analogs of α-melanocyte-stimulating hormone (α-MSH) (which binds to MC1R as seen in Figure 4) have been developed and extensively tested, and have demonstrated the effect of enhancing the repair of DNA photoproducts and reducing reactive oxygen species (ROS) generation and apoptosis in ultraviolet radiation-irradiated melanocytes [34]. Activation of MC1R has been shown to produce eumelanin through an intracellular cascade reaction, as outlined in Figure 5 [41]. In this cascade, the agonist α-MSH binds to MC1R in the setting of solar irradiation, which then induces dissociation of the G α-subunit. Adenyl cyclase is activated, leading to an accumulation of intracellular cAMP. Protein kinase A is then activated by this cAMP, thereby phosphorylating a variety of downstream effector pathways, including induction of the CREB and Mitf transcription factor networks, ultimately leading to the expression of tyrosinase and other enzymes involved in melanin synthesis [41].

**Figure 6:**
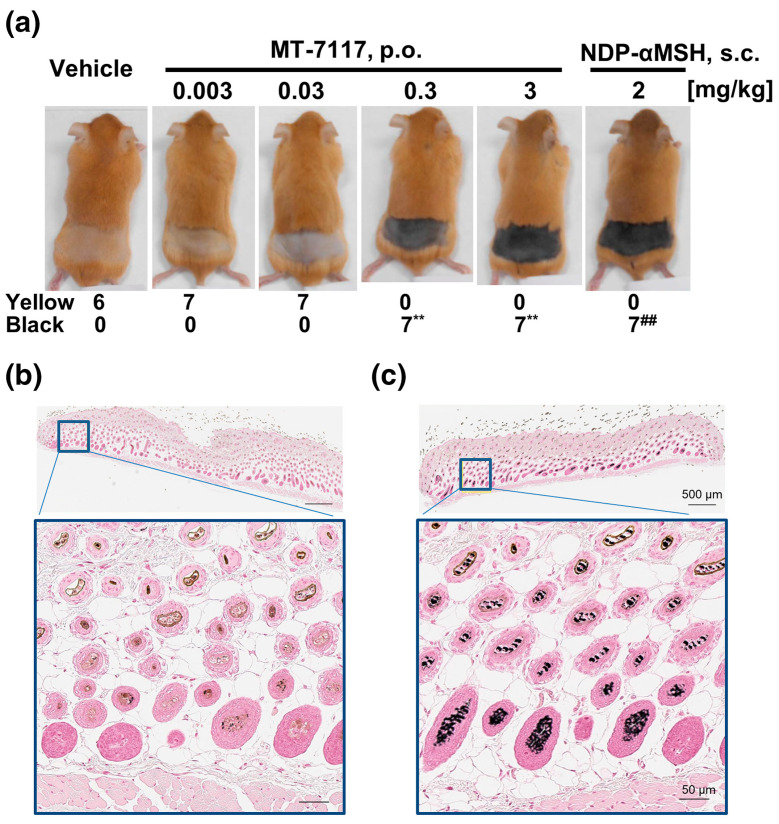
Effects of Dersimelagon [MT-7117] or Afamelanotide [NDP-αMSH] on the coat color darkening of Ay/a mice. MT-7117 and NDP-αMSH were administered for 6 days, dorsal coat was shaved, and newly grown coat color was assessed as yellow or black. (**a**) Representative image of each group on day 6 and the number of mice with the emergent coat color determined as yellow or black. ## *p* < 0.01 versus vehicle by Fisher’s exact test, ** *p* < 0.01 versus vehicle by Fisher’s exact test with multiplicity adjusted using fixed sequence procedure. (**b**) Representative image of Fontana-Masson staining of dorsal skin in the vehicle-treated group on day 6 with hair root pigment as yellow. (**c**) Representative image of Fontana-Masson staining of dorsal skin in the vehicle-treated group on day 6 with hair root pigment as black [38].

Dersimelagon increases cAMP levels in a dose-dependent manner. Pharmacokinetic studies revealed that dersimelagon was able to produce Emax values similar to those of αMSH, suggesting that they have similar agonistic activity on variants of MC1R [38]. Plasma concentrations of the drug were analyzed and revealed a greater-than-dose-proportional increase in serum levels of dersimelagon with increased oral dosing in animal studies. Oral administration of dersimelagon produced the MC1R agonistic activity levels necessary to induce skin pigmentation in murine models (Figure 6). As outlined in Figure 6, MC1R agonists increased melanin production in the hair root in a dose-dependent manner; 1 mg/kg was found to be the minimally effective dose for clinically significant melanin production. Over six consecutive days, dersimelagon (MT-7117) was administered orally and afamelanotide (NDP a-MSH) was administered subcutaneously. As seen in Figure 6c, MT-7117 elicited synthesis of eumelanin, rather than pheomelanin. While dersimelagon is selective for MC1R, afamelanotide non-selectively binds MC1R, MC3R, and other melanocortin receptors [24,38]. Perhaps nausea often produced by afamelanotide may be attributed to its binding to MC3R and downstream effects [43].

## 5. Key Clinical Trial Results for Dersimelagon

The first-in-human phase 1 trial conducted for dersimelagon enrolled 144 healthy participants (143 completed the trial) and demonstrated an acceptable safety profile in these patients [44]. Thirty-four of these patients received a placebo, with the remaining patients receiving at least one dose of dersimelagon (doses ranged from 1 to 600 mg). Additionally, 36 of the dersimelagon patients received multiple doses ranging from 30 to 450 mg. There were few treatment-emergent adverse events (TEAEs) with no death or serious AE reported. Most symptoms reported were mild to moderate in severity. However, overall, patients receiving multiple doses of dersimelagon had more TEAEs. The most common TEAEs reported were related to skin pigmentation, including lentigo, skin hyperpigmentation, and melanocytic nevi (two cases were severe but non-malignant). Additionally, there were no statistically significant effects of age or race on the pharmacokinetics of dersimelagon; however, increased exposure was observed in females compared to males, which was attributed to differences in the body weight generally observed in male versus female participants [44]. 

**Figure 7:**
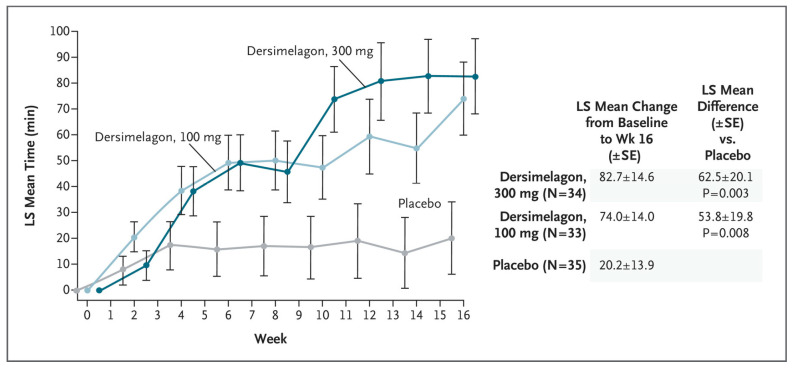
Primary End Point: Time to First Prodromal Symptom in an intention-to-treat population [13].

A phase 2 randomized, double-blind, placebo-controlled study set out to evaluate the safety and efficacy of dersimelagon concerning the time to prodromal symptoms (TTP) and severity of symptoms associated with sunlight exposure for patients with EPP or XLP (Figure 7) [13]. The population included both males and females with confirmed diagnoses of EPP between the ages of 18–75 who were assigned in a 1:1:1 ratio to receive a placebo, dersimelagon 100 mg, or dersimelagon 300 mg once daily for 16 weeks. Exclusion criteria included those with acute or chronic renal disease, pregnancy or lactation, clinically significant hepatobiliary disease, history of non-EPP photodermatoses, excessive alcohol intake, melanoma, psychiatric disease, treatment with phototherapy, antioxidant agents, or afamelanotide within 3 months before trial. Of the 102 randomized patients (93 with EPP and 9 with XLP), a statistically significant increase in the least mean change-square in TTP from baseline to week 16 was noted, with the 100 mg group being 74 ± 14 min (*p* = 0.008) and the 300 mg group being 82.7 ± 14.6 min (*p* = 0.003) v placebo (20.2 ± 13.9 min). Secondary endpoints, including incidence rate and total number of phototoxic pain events, and total duration of sunlight exposure without prodromal symptoms, all demonstrated improvement, regardless of dersimelagon dose [45].

Dersimelagon was well tolerated overall. Most adverse events were mild to moderate in severity and resolved within the trial period. The groups evaluated were placebo, dersimelagon 100 mg, and dersimelagon 300 mg. The most frequently reported adverse effect was nausea (12%, 15%, and 46%, respectively), followed by headache (18%, 18%, and 29%), freckles (0%, 15%, and 31%), and skin hyperpigmentation (0%, 9%, and 31%). The one serious adverse event that led to discontinuation by a patient was an anaphylactic reaction that was deemed by the investigators to be unrelated to dersimelagon [45].

Post hoc analysis of this phase 2 trial evaluated patients’ perspectives of their quality of life and the role of geographic location within different seasons (given anticipated changes in UV light intensity). Two subgroups (Subgroup 1: Spring/Summer; Subgroup 2: Fall/Winter) were evaluated in both the northern and southern United States. Patients taking dersimelagon were found to have a statistically significant increase in the time to symptoms development with sun exposure in both subgroups and geographical locations. For example, in Subgroup 1, when compared to placebo, the time to symptoms increased by 38.0 min with dersimelagon 100 mg and by 39.7 min with dersimelagon 300 mg. By assessing this metric across all groups, there was a significant increase when compared with placebo, rising by 21.9 min with dersimelagon 100 mg and by 22.1 min with dersimelagon 300 mg (*p* = 0.032; *p* = 0.004, respectively). Additionally, an online questionnaire assessing quality of life was completed by 75 of the 102 patients enrolled. Of those taking dersimelagon 100 mg, 57.6% rated their EPP as “very much better” and 69.7% rated they were “much more often” able to be outside at the end of the study. Likewise, among those who used dersimelagon 300 mg, positive ratings were given by 48.6%. [46].

## 6. Limitations 

As outlined above, most adverse events associated with the use of dersimelagon were mild to moderate in severity and resolved within the trial period. Participation in the trial was discontinued by four patients in the placebo group (voluntary withdrawal), two patients in the 100 mg dersimelagon group (one for nonadherence and one for a serious adverse event), and four patients in the 300 mg dersimelagon group (one voluntary withdrawal, two withdrawals for adverse events, and one withdrawal for clinically significant lichen planus found during nevi assessment). The serious adverse event that led to discontinuation by one patient in the 100 mg dersimelagon group was an anaphylactic reaction that was deemed by the investigators to be unrelated to dersimelagon [45]. Additionally, there was incomplete blinding in this trial due to increases in skin pigmentation when taking dersimelagon, which allowed many subjects to correctly guess if they were assigned to the active drug group.

In the phase 2 trial, limitations included a lack of formal statistical power analysis, although such limitations are common in studies of rare diseases such as EPP/XLP [13]. 

Continuing concerns surrounding the chronic use of dersimelagon primarily involve the long-term safety and tolerability of the drug. A concern arises regarding the possible stimulation of abnormal melanocytes in the human body, given that melanoma tumors comprise a mixture of cells with only a subset expressing MC1R. There have been some experiments suggesting reduced MC1R expression may contribute to melanoma as melanin cells become less differentiated. Thus, an increase in MC1R expression could portend a possible lower risk for subsequent melanoma development, as the authors of these studies suggest [47,48]. We anticipate that these provocative results will require ongoing study. It should be noted that a literature review conducted in 2013 on afamelanotide evaluated concerns about its potential to contribute to the malignant transformation of melanocytes [49]. In this review, no documented cases of malignancy due to afamelanotide were noted, and instead the authors suggested that it may inhibit melanoma cell proliferation [49]. Later study of the effects of dersimelagon in preclinical in vitro studies on five separate human melanoma cell lines and found that dersimelagon did not affect the proliferation of these cell lines [38]. Additionally, another study showed that α-MSH did not stimulate increased proliferation or invasion of malignant cells in a nonclinical in vitro study [48].

To further evaluate the potential for drug-induced liver injury, more subjects observed for longer treatment times are needed. In cross-sectional studies of patients with EPP/XLP, up to 25% had a reported elevation in liver enzymes. A rare, yet severe and rapidly progressive form of liver involvement that can lead to acute liver failure, known as protoporphyric hepatopathy, occurs in a small proportion (2–5%) of patients with protoporphyria. It is suspected that patients with EPP and XLP with underlying genetic disease or superimposed etiologies (biliary tract disease, viral hepatitis, autoimmune conditions, alcohol, etc.) may have an increased likelihood of EPP-related liver failure, although this is not yet well-defined [17,50,51].

## 7. Conclusions

Strides are being made to gain insight into the true prevalence of EPP/XLP, which is likely under-recognized. As such, there may be an underappreciation for the devastating effects on patients with these conditions. The only current EMA- or FDA-approved therapy, afamelanotide, is subcutaneously injected. Additional uncertainties related to receiving this therapeutic have been posed, including the fact that only few centers that have been provided with afamelanotide, the expertise required for administration, and significant cost concerns. Dersimelagon offers hope for an alternative agent to improve patient quality of life. Dersimelagon thus far has demonstrated an acceptable safety profile, with ease of once-daily oral dosage. Ongoing studies will provide additional insights into expanding the treatment options for those suffering from EPP/XLP.

## Figures and Tables

**Table 1:** Overview of Cutaneous Porphyrias and The Acute Porphyrias in which Cutaneous Manifestations may Occur. Abbreviations used: AD, autosomal dominant; ALA, 5-aminolevulinic acid; AR, autosomal recessive; ESRD, end-stage renal disease; PBG, porphoblilinogen; ↓, decreasing.

The Protoporphyrias				
Disease	Inheritance	Enzyme/Genetic Abnormality	Clinical	Comment
Erythropoietic protoporphyria (EPP)	Autosomal Recessive (AR)	FECH (↓ activity)	Acute Photosensitivity—pain, redness, swelling Rarely, general paresis in setting of liver failure or after liver transplant	Most common is missense or nonsense mutation on 1 allele and IVS3-48T>C leading to ↓ expression on the other allele
X-linked protoporphyria (XLP)	X-linked	ALA-synthase-2 (Gain-of-function)	Acute Photosensitivity—pain, redness, swelling Rarely, general paresis in setting of liver failure of after liver transplant	Most common are deletions in Exon 11
The Uroporphyrias				
Porphyria cutanea tarda (PCT) type 1 (acquired)	None —acquired	Hepatic uroporphyrinogen III decarboxylase (UROD)	Chronic blistering and bullae formation of sun-exposed skin; chronic actinic damage	Major risk factors: alcohol, estrogen, iron, HCV
PCT—type 2 (familial)	Autosomal recessive (AR)	UROD	Chronic blistering and bullae formation of sun-exposed skin; chronic actinic damage	50% ↓ in enzyme activity insufficient to cause clinical disease; also need other risk factors (as above)
Hepato-erythropoietic porphyria (HEP)	AR homozygous or compound heterozygous	UROD	Severe blistering and bullae formation; hypertrichosis—occurring early in life- Infancy/childhood	Severe deficiency, leading to severe disease early in life
Congenital erythropoietic porphyria (CEP)	AR homozygous or compound heterozygous	UROS [aka URO3 Co-synthase]	Severe blistering and bullae formation; hypertrichosis—occurring early in life	Severe deficiency, with severe disease early in life; may also occur due to mutations in abnormal clones of developing red blood cells
Acute Porphyrias +/− Cutaneous Features				
Hereditary coproporphyria (HCP)	Autosomal dominant (AD)	Coproporphyrinogen oxidase (CPOX)	Blisters and bullae as in PCT + Acute attacks of generalized, poorly Localized abdominal pain and variable other neurological features	Cutaneous features rare
Variegate porphyria (VP)	AD	Protoporphyrinogen oxidase (PPOX)	Blisters and bullae as in PCT + Acute attacks of generalized, poorly Localized abdominal pain and variable other neurological features	Cutaneous features common, +/− symptoms of acute porphyria
Acute intermittent Porphyria (AIP)	AD	Hydroxymethylbilane Synthase [HMBS, aka PBG deaminase]	Acute attacks of generalized, poorly Localized abdominal pain and variable other neurological features + rarely Blisters and bullae as in PCT, HEP	Cutaneous features may occur in the setting of highly active AIP [homozygous or compound heterozygous severe HMBS deficiency] with very high ALA, PBG, and porphyrin overproduction and/or with ESRD leading to inability to excrete uroporphyrin

**Table 2:** Summary of Pharmacokinetic Parameters of Dersimelagon following oral dosing in healthy adults [18].

Pharmacokinetic Parameter	Plasma Total Radioactivity	Whole Blood Total Radioactivity
C_max_ (ng/mL) ^a^	432.20 (151.20)	219.00 (72.20)
T_max_ (h)	2.00 (2.01)	2.00 (2.01)
AUC_0−t_ (ng·h/mL) ^a^	3754.0 (1163.00)	1158.00 (440.00)
AUC_0−∞_ (ng·h/mL) ^a^	4462.00 (1063.00)	3311.00 (2268.00)
t_1/2_ (h)	12.70 (5.32)	15.73 (21.43)
K_el_ (/h)	0.06 (0.02)	0.11 (0.07)

Note: Arithmetic mean (SD) is presented for all variables except T_max_, for which medial (range) is presented. Abbreviations: AUC, area under the concentration-time curve; AUC_0−∞_, AUC from time 0 extrapolated to infinity; AUC_0−t_, AUC from time 0 to the time of the last quantifiable concentration; C_max_, maximum observed concentration; h, hours; K_el_, terminal elimination rate constant; t_1/2_, apparent terminal elimination half-life; T_max_, time to C_max_, ^a^ Units for total radioactivity AUCs and C_max_ are ng equivalents·h/mL and ng equvalents/mL, respectively.

**Table 3:** Comparison of Dersimelagon (MT-7117) and Afamelanotide (NDP-αMSH) binding affinities to four MCR [38].

	Ki Value for Receptor Binding (nmol/L)	
Human Recombinant Receptor	MT-7117	NDP-α MSH
MC1R	2.26	0.028
MC3R	1420	0.17
MC4R	32.9	0.20
MC5R	486	0.21

## Data Availability

Research data was not collected in this review.

## References

[1] Heerfordt I.M., Philipsen P.A., Wulf H.C. (2022). Bringing the gentle properties of daylight photodynamic therapy indoors: A systematic review of efficacy and safety. Photodiagn. Photodyn. Ther..

[2] Heerfordt I.M., Lerche C.M., Wulf H.C. (2022). Cimetidine for erythropoietic protoporphyria. Photodiagn. Photodyn. Ther..

[3] Lim H.W. (1989). Mechanisms of phototoxicity in porphyria cutanea tarda and erythropoietic protoporphyria. Immunol. Ser..

[4] Bottomley S.S., Tanaka M., Everett M. (1975). Diminished erythroid ferrochelatase activity in protoporphyria. J. Lab. Clin. Med..

[5] Bonkowsky H.L., Bloomer J.R., Ebert P.S., Mahoney M.J. (1975). Heme synthetase deficiency in human protoporphyria. Demonstration of the defect in liver and cultured skin fibroblasts. J. Clin. Investig..

[6] Levy C. (2023). Overview of liver involvement in patients with erythropoietic protoporphyria. Gastroenterol. Hepatol..

[7] Todd D. (1994). Erythropoietic protoporphyria. Br. J. Dermatol..

[8] Brancaleoni V., Balwani M., Granata F., Graziadei G., Missineo P., Fiorentino V., Fustinoni S., Cappellini M.D., Naik H., Desnick R. (2016). X-chromosomal inactivation directly influences the phenotypic manifestation of X-linked protoporphyria. Clin. Genet..

[9] Balwani M., Doheny D., Bishop D.F., Nazarenko I., Yasuda M., Dailey H.A., Anderson K.E., Bissell D.M., Bloomer J., Bonkovsky H.L. (2013). Porphyrias Consortium of the National Institutes of Health Rare Diseases Clinical Research Network: Loss-of-function ferrochelatase and gain-of-function erythroid-specific 5-aminolevulinate synthase mutations causing erythropoietic protoporphyria and x-linked protoporphyria in North American patients reveal novel mutations and a high prevalence of X-linked protoporphyria. Mol. Med..

[10] Balwani M. (2019). Erythropoietic protoporphyria and X-linked protoporphyria: Pathophysiology, genetics, clinical manifestations, and management. Mol. Genet. Metab..

[11] Di Pierro E., Granata F., De Canio M., Rossi M., Ricci A., Marcacci M., De Luca G., Sarno L., Barbieri L., Ventura P. (2022). Recognized and emerging features of erythropoietic and X-linked protoporphyria. Diagnostics.

[12] Sandberg S., Talstad I., Hovding G., Bjelland N. (1983). Light-induced release of protoporphyrin, but not of zinc protoporphyrin, from erythrocytes in a patient with greatly elevated erythrocyte protoporphyrin. Blood.

[13] Balwani M., Bonkovsky H.L., Levy C., Anderson K.E., Bissell D.M., Parker C., Takahashi F., Desnick R.J., Belongie K. (2023). Dersimelagon in Erythropoietic Protoporphyrias. N. Engl. J. Med..

[14] Dickey A.K., Quick C., Ducamp S., Zhu Z., Feng Y.C.A., Naik H., Balwani M., Anderson K.E., Lin X., Phillips J.E. (2021). Evidence in the UK Biobank for the underdiagnosis of erythropoietic protoporphyria. Genet. Med..

[15] Elder G., Harper P., Badminton M., Sandberg S., Deybach J. (2013). The incidence of inherited porphyrias in Europe. J. Inherit. Metab. Dis..

[16] Schulenburg-Brand D., Katugampola R., Anstey A.V., Badminton M.N. (2014). The cutaneous porphyrias. Dermatol. Clin..

[17] Dickey A.K., Naik H., Keel S.B., Levy C., Beaven S.W., Elmariah S.B., Erwin A.L., Goddu R.J., Hedstrom K., Leaf R.K. (2023). Porphyrias Consortium of the Rare Diseases Clinical Research Network. Evidence-based consensus guidelines for the diagnosis and management of erythropoietic protoporphyria and X-linked protoporphyria. J. Am. Acad. Dermatol..

[18] Tsuda M., Ogawa K., Endou T., Goto T., Ogasawara Y., Ogasawara A. (2023). Absorption, metabolism, and excretion of [^14^C] dersimelagon, an investigational oral selective melanocortin 1 receptor agonist, in preclinical species and healthy volunteers. Pharmacol. Res. Perspect..

[19] Kaye E.T., Levin J.A., Blank I.H., Arndt K.A., Anderson R.R. (1991). Efficiency of opaque photoprotective agents in the visible light range. Arch. Dermatol..

[20] Sivaramakrishnan M., Woods J., Dawe R. (2014). Narrowband ultraviolet B phototherapy in erythropoietic protoporphyria: Case series. Br. J. Dermatol..

[21] Mathews-Roth M.M., Pathak M.A., Fitzpatrick T.B., Harber L.C., Kass E.H. (1974). β-Carotene as an oral photoprotective agent in erythropoietic protoporphyria. JAMA.

[22] Mathews-Roth M.M. (1984). Treatment of erythropoietic protoporphyria with beta-carotene. Photo-Dermatology.

[23] Mathews-Roth M.M., Pathak M.A., Fitzpatrick T.B., Harber L.H., Kass E.H. (1977). Beta carotene therapy for erythropoietic protoporphyria and other photosensitivity diseases. Arch. Dermatol..

[24] Minder E. (2010). Afamelanotide, an antagonistic analog of alpha-melanocyte-stimulating hormone, dermal phototoxicity of erythropoietic protoporphyria. Expert Opin. Investig. Drugs.

[25] Krook G., Haeger-Aronsen B. (1982). Beta-carotene in the treatment of erythropoietic protoporphyria. A short review. Acta Derm. Venereol. Suppl..

[26] Collins P., Ferguson J. (1995). Narrow-band UVB (TL-01) phototherapy: An effective preventative treatment for the photodermatoses. Br. J. Dermatol..

[27] Bijlmer-Iest J.C., De La Faille H.B., Van Asbeck B.S., Van Hattum J., Van Weelden H., Marx J.J., Koningsberger J.C. (1993). Protoporphyrin photosensitivity cannot be attenuated by oral N-acetylcysteine. Photodermatol. Photoimmunol. Photomed..

[28] McGuire B.M., Bonkovsky H.L., Carithers R.L., Chung R.T., Goldstein L.I., Lake J.R., Lok A.S., Potter C.J., Rand E., Voigt M.D. (2005). Liver transplantation for erythropoietic protoporphyria liver disease. Liver Transpl..

[29] Corbett M.F., Herxheimer A., Magnus I.A., Ramsay C.A., Kobza-Black A. (1977). The long-term treatment with beta-carotene in erythropoietic protoporphyria: A controlled trial. Br. J. Dermatol..

[30] Wensink D., Wagenmakers M.A., Langendonk J.G. (2021). Afamelanotide for prevention of phototoxicity in erythropoietic protoporphyria. Expert Rev. Clin. Pharmacol..

[31] European Medicines Agency EU/3/22/2585—Orphan Designation for Treatment of Erythropoietic Protoporphyria. https://www.ema.europa.eu/en/medicines/human/orphan-designations/eu-3-22-2585.

[32] Minder A.-E., Schneider-Yin X., Zulewski H., Minder C.E., Minder E.I. (2023). Afamelanotide is associated with dose-dependent protective effect from liver damage related to erythropoietic protoporphyria. Life.

[33] Levine N., Sheftel S.N., Eytan T., Dorr R.T., Hadley M.E., Weinrach J.C., Ertl G.A., Toth K., McGee D.L., Hruby V. (1991). Induction of skin tanning by subcutaneous administration of a potent synthetic melanotropin. JAMA.

[34] García-Borrón J.C., Abdel-Malek Z., Jiménez-Cervantes C. (2014). MC1R, the cAMP pathway, and the response to solar UV: Extending the horizon beyond pigmentation. Pigment Cell Melanoma Res.

[35] Lane A.M., McKay J.T., Bonkovsky H.L. (2016). Advances in the management of erythropoietic protoporphyria—The role of afamelanotide. Appl. Clin. Genet..

[36] Swetlitz I. (2023). Clinuvel Limits the Availability of Scenesse for Rare Genetic Disease. Bloomberg.

[37] Yao J.-F., Yang H., Zhao Y.-Z., Xue M. (2018). Metabolism of peptide drugs and strategies to improve their metabolic stability. Curr. Drug Metab..

[38] Suzuki T., Kawano Y., Matsumoto A., Kondo M., Funayama K., Tanemura S., Miyashiro M., Nishi A., Yamada K., Tsuda M. (2022). Melanogenic effect of dersimelagon (MT-7117), a novel oral melanocortin 1 receptor agonist. Skin Health Dis..

[39] Ogawa K., Ide R., Belongie K., Tsuda M., Kawanishi H., Teng R., Ogasawara A. (2023). The oral bioavailability and effect of various gastric conditions on the pharmacokinetics of dersimelagon in healthy adult volunteers. Clin. Pharmacol. Drug Dev..

[40] Mun Y., Woo K., Dongyun S. (2023). Melanocortin 1 Receptor (MC1R): Potentials as therapeutic targets. Int. J. Mol. Sci..

[41] Horrell E.M.W., Boulanger M.C., D’orazio J.A. (2016). Melanocortin 1 Receptor: Structure, function, and regulation. Front. Genet..

[42] Dorr R.T., Ertl G., Levine N., Brooks C., Bangert J.L., Powell M.B., Humphrey S., Alberts D.S. (2004). Effects of a superpotent melanotropic peptide in combination with solar UV radiation on tanning of the skin in human volunteers. Arch. Dermatol..

[43] Langendonk J.G., Balwani M., Anderson K.E., Bonkovsky H.L., Anstey A.V., Bissell D.M., Bloomer J., Edwards C., Neumann N.J., Parker C. (2015). Afamelanotide for erythropoietic protoporphyria. N. Engl. J. Med..

[44] Ogasawara A., Ogawa K., Ide R., Ikenaga Y., Fukunaga C., Nakayama S., Tsuda M. (2023). Results from a first-in-human study of dersimelagon, an investigational oral selective MC1R agonist. Eur. J. Clin. Pharmacol..

[45] Belongie K., Takahashi F., Martin S. (2002). 34420 Perspective of patients with erythropoietic protoporphyria treated with dersimelagon, a selective melanocortin-1 receptor agonist: Results of the ENDEAVOR study exit questionnaire. J. Am. Acad. Dermatol..

[46] Belongie K., Takahashi F., Mukai S., Endou M., Desnick R. (2022). 33323 Dersimelagon, a melanocortin-1 receptor agonist, maintains efficacy regardless of seasons in subjects with erythropoietic protoporphyria: Results from a post hoc analysis of phase 2 clinical trial data. J. Am. Acad. Dermatol..

[47] Murata J. (1997). Alpha-melanocyte-stimulating hormone blocks invasion of reconstituted basement membrane (Matrigel) by murine B16 melanoma cells. Invasion Metastasis.

[48] Kondo M., Suzuki T., Kawano Y., Kojima S., Miyashiro M., Matsumoto A., Kania G., Błyszczuk P., Ross R.L., Mulipa P. (2022). Dersimelagon, a novel oral melanocortin 1 receptor agonist, demonstrates disease-modifying effects in preclinical models of systemic sclerosis. Arthritis Res. Ther..

[49] Fabrikant J., Touloei K., Brown S. (2013). A review and update on melanocyte stimulating hormone therapy: Afamelanotide. J. Drugs Dermatol..

[50] Singal A.K., Parker C., Bowden C., Thapar M., Liu L., McGuire B.M. (2014). Liver transplantation in the management of porphyria. Hepatology.

[51] Bonkovsky H.L., Schned A.R. (1986). Fatal liver failure in protoporphyria: Synergism between ethanol excess and the genetic defect. Gastroenterology.

